# Editorial: Aging Research and Practices in Malaysia

**DOI:** 10.3389/fpubh.2022.948822

**Published:** 2022-06-30

**Authors:** Siti Anom Ahmad, Pin Maw Tan, Devinder Kaur Ajit Singh, Rahimah Ibrahim, Pei-Lee Teh, Tengku Aizan Hamid

**Affiliations:** ^1^Malaysian Research Institute on Ageing, Universiti Putra Malaysia, Serdang, Malaysia; ^2^Ageing and Age-Associated Disorders Research Group, Department of Medicine, Faculty of Medicine, Universiti Malaya, Kuala Lumpur, Malaysia; ^3^Center for Healthy Aging & Wellness, Faculty of Health Sciences, Universiti Kebangsaan Malaysia, Kuala Lumpur, Malaysia; ^4^School of Business, Gerontechnology Lab, Monash University, Bandar Sunway, Malaysia

**Keywords:** aging, healthy aging, wellbeing, best practices, technology

Malaysia is a rapidly aging upper middle-income nation with the population aged 65 years and over expected to increase 3-fold within the next 20 years. With its universities rapidly ascending international rankings, the research output within this country is also increasing at an unprecedented rate. As a result, our Research Topic on “*Aging Research and Practices in Malaysia*” has generated a great deal of interest and boasts 22 articles in total. The articles provide a good representation of the types of research ongoing in Malaysia to address the pressing issue of population aging.

The clinical research areas covered within this Research Topic included cognitive frailty, stroke, osteoarthritis (OA) and infection. Contributors included a team from the Universiti Kebangsaan Malaysia which published their protocol on multimodal intervention for cognitive frailty -WE-RISE (Murukesu et al.). Mohamad Fuad et al. conducted a controlled study using serial cognitive assessments after stroke confirming the high risk of cognitive decline after stroke. Controversially, Mat et al. found that the presence of OA symptoms was protective against falls after muscle strength is accounted for. The perspectives of older persons with OA toward the Enabling Self-management and Coping with Arthritic Pain using Exercise (ESCAPE-Pain) interventional programme were evaluated with positive responses recorded (Hasan et al.). Akhtar et al. found that treatment outcome in urinary tract infection is influenced by gender, polypharmacy and comorbidities. The above series of articles demonstrates a wide spectrum of research methods ranging from qualitative to quantitative design, observational to interventional and cross-sectional to prospective now being utilized in clinical studies in older adults.

Falls related research was heavily represented within this issue. The studies focused on home hazards among stroke survivors (Ainuddin et al.), falls risk in older persons (Ashari et al.), fear of falls (FOF) (Romli et al.), falls incidence recording in research (Romli et al.) and ankle muscle function in older persons (Perera et al.). Generally, the findings showed that there is a lack of research pertaining to home environment risk assessment and intervention in stroke survivors. Factors associated with FOF include limitations in daily functional activities but not home hazards. Instability during turning, visual impairment and back pain were the highlighted falls risk factors in Malaysian older adults. In addition, decreased toe clearance with limited knee flexion and ankle dorsiflexion was established using gait analysis in a simulated study in older persons with falls. In a pilot study testing community-based strengthening exercises for older persons with frailty demonstrated improvement in both upper and lower limb muscles post-intervention (Raja Adnan et al.). Lastly, one of the novel recommendations from this issue was to consider both retrospective recall and prospective recording of falls incidence using falls calendars in research.

Psychological research covered in this topic comprises prospective memory training and knowledge, attitude and practices on salt intake whereas socio-economic research is based on social factors such as dependency ratio, social networks and dementia caregiving. The study by Haron et al. highlighted the importance of knowledge on salt intake along with the aptitude to learn within one's social context to reduce hypertension among older persons. Multicomponent prospective memory training, which is tailored to older people, is emphasized by Farzin et al. as a strategy to promote independence and wellbeing. Hamid et al. found that social networks may have a stronger influence on older adults' mental health compared to their living arrangements. Goodson et al. stressed the importance of dementia awareness and caregiver training highlighting the issues surrounding the dementia care system which is difficult to navigate. Active aging and labor force participation is proposed by Mohd et al. as strategies to mitigate the negative impact of the old-age dependency ratio on economic growth. Psychological studies underscore the role of learning theories in behavior modification and healthy aging. The authors' contributions demonstrate the importance of psychological and socio-economic factors to older people's health and wellbeing.

In this Research Topic, how technology could support various health issues related to aging has been covered. Falls are related to instability either permanent or transient and to have a better understanding of physical limitations and visual issues among older persons, research using aging simulation suits could enhance health advocacy among health practitioners (Lee and Teh). Perception and expectations analysis toward the use of fall detection devices have shown definite interest in using these devices with user friendly, affordable, and accurate features (Rahman et al.). Research on muscles affecting minimum toe clearance during gait could also reduce risk of falls, as with aging the muscles would weaken and stiffen, coupled with reduced motion join range (Perera et al.). The findings of a scoping review concluded that the technological interventions in stroke rehabilitation had a positive impact on stroke rehabilitation (Selamat et al.).

Ng et al. has implemented a Health Care of Elderly course to public university students to look at their attitude, self-perceived competence and interest toward geriatric medicine as a career and found there are positive interests after the course. They also highlighted the importance to increase preparedness in managing frail older adults with multimorbidities. To address issues of limited health screening resources for older persons, Alex et al. has conducted a self-administered online survey in urban community dwelling to screen geriatric syndromes and conclude it is feasible to do it online.

The explosion of aging research in Malaysia has been apparent over the past 5 years. This Research Topic showcases the breadth of aging research in Malaysia associated with its rapid population aging that affects individuals, families and society. The published articles also demonstrated extensive inter-disciplinary collaborations within health, economy, social and technology areas. Through this topic, an understanding about aging Malaysia: research and practice has no doubt been enhanced.

## Author Contributions

PT has prepared the initial draft of the Editorial. SA has compiled the article and was reviewed by all authors again, before approving the final version. All authors have made substantial contributions in this writing.

## Conflict of Interest

The authors declare that the research was conducted in the absence of any commercial or financial relationships that could be construed as a potential conflict of interest.

## Publisher's Note

All claims expressed in this article are solely those of the authors and do not necessarily represent those of their affiliated organizations, or those of the publisher, the editors and the reviewers. Any product that may be evaluated in this article, or claim that may be made by its manufacturer, is not guaranteed or endorsed by the publisher.

